# Enhanced therapeutic efficacy of anti-PD-1 blockade by targeting LAMP2A inhibits lysosomal degradation of STING and TBK1

**DOI:** 10.7150/thno.103426

**Published:** 2025-02-18

**Authors:** Xueying Wang, Diekuo Zhang, Jiaqing Xiao, Lei Wang, Junrong Wang, Xiaoqiao Cui, Jiaqi Tan, Yong Liu, Susheng Miao

**Affiliations:** 1 Department of Otolaryngology Head and Neck Surgery, Xiangya Hospital, Central South University, No. 87 Xiangya Road, Changsha 410008, Hunan, The People's Republic of China.; 2 Otolaryngology Major Disease Research Key Laboratory of Hunan Province, No. 87 Xiangya Road, Changsha 410008, Hunan, The People's Republic of China.; 3 Clinical Research Center for Laryngopharyngeal and Voice Disorders in Hunan Province, No. 87 Xiangya Road, Changsha 410008, Hunan, The People's Republic of China.; 4 Institute of Heilongjiang Province Center for Disease Control Disinfection and Infection, Harbin 150081, Heilongjiang, The People's Republic of China;; 5 Department of Otorhinolaryngology Head and Neck Surgery, Harbin Medical University Cancer Hospital, Harbin 150081, Heilongjiang, The People's Republic of China.; 6 National Clinical Research Center for Geriatric Disorders (Xiangya Hospital), Changsha 410008, Hunan, The People's Republic of China.

**Keywords:** CMA, LAMP2A, STING, TBK1, PD-1

## Abstract

**Rationale:** LAMP2A is a key translocase in chaperone-mediated autophagy (CMA), and the STING/TBK1 axis is crucial in antitumor immunity. This study explored the complex mechanisms by which CMA regulates the STING/TBK1 degradation and whether targeting LAMP2A could enhance the efficacy of PD-1 monoclonal antibodies.

**Methods:** The expression of STING and TBK1 was detected after treatment with various inhibitors of protein degradation pathways. Confocal microscopy was used to detect the localization of STING and TBK1 in lysosomes. R software was used to analyze LAMP2A expression and prognosis. The biological function of LAMP2A was examined by *in vitro* and *in vivo* experiments.

**Results:** Through *in vitro* and *in vivo* experiments and a review of clinical specimens, we identified STING/TBK1 as a novel substrate of CMA. Downregulation of LAMP2A enhanced IFNβ production and cellular antiviral response by inhibiting CMA-mediated degradation of STING and TBK1. Based on these observations, further *in vivo* experiments confirmed that the LAMP2A loss combined with PD-1 monoclonal antibodies significantly stimulated the activation of infiltrating CD8+ T cells, thereby inhibiting tumor growth. Also, non-responder head and neck squamous cell carcinoma (HNSCC) patients undergoing neoadjuvant immuno-chemotherapy had higher levels of LAMP2A and lower levels of PD-L1 expression in their tumor tissues.

**Conclusions:** Our study has revealed a prospective combination therapy, in which diclofenac functioning as a LAMP2A inhibitor, enhances the anti-tumor efficacy of PD-1 monoclonal antibody by inhibiting the degradation of STING and TBK1.

## Introduction

HNSCC is a highly heterogeneous malignancy, exhibiting aberrant inactivation of various tumor-suppressive signaling pathways at variable frequencies 1. In recent years, immune checkpoint inhibitors, such as the anti-PD-1 monoclonal antibody, have evolved to treat advanced-stage HNSCC, which typically exhibits a scarcity of cytotoxic immune cells coupled with the infiltration of immunosuppressive cellular phenotypes, including regulatory T cells and myeloid-derived suppressor cells (MDSCs). These immunosuppressive characteristics pose a formidable impediment to the antitumor response 2, underscoring the pressing need to explore the interplay between intrinsic events within tumor cells and host immune responses in HNSCC. The crucial regulatory factor, stimulator of interferon genes, with its downstream pathway involving TBK1-IRF3, plays a significant role in immune surveillance and immune-mediated cytotoxicity against tumors, inhibiting tumor cell survival, proliferation, and migration 3-5. The ubiquitin-proteasome system or autophagy, which contributes to the degradation of STING and TBK1, has been shown to favor the growth of different tumor types 6-9. Induction of autophagy is a primordial function of the cGAS-STING pathway, and the degradation pathways of STING and TBK1 continue to draw attention 10. Notably, Wei *et al.* found that STING recruits WIPI2 for autophagosome formation, which is essential for the clearance of cytoplasmic DNA and the subsequent attenuation of the cGAS-STING signaling. This discovery was crucial for understanding the molecular mechanisms by which the cGAS-STING pathway induces autophagosome formation 11-13.

Autophagy is an orchestrated catabolic mechanism that enables cells to endure adverse conditions, such as hypoxia, nutrient deprivation, or cytotoxic substances. Studies have demonstrated that autophagy, among other physiological and pathological processes 14, plays a significant role in cellular homeostasis, aging, immunity, tumorigenesis, and neurodegenerative diseases. Based on the nature of the cargo and the mode of transportation, autophagy can be classified into three types: 1. Macroautophagy involves the formation of double-membrane structures called autophagosomes, which encapsulate intracellular materials and eventually fuse with lysosomes. Typically, autophagy is referred to in the context of macroautophagy. 2. Microautophagy occurs through the direct engulfment of specific organelles by the lysosomal or vacuolar surface deformation. And 3. CMA represents a specific form of autophagy wherein select proteins are transported to lysosomes for degradation with the assistance of molecular chaperones.

Proteins bearing the KFERQ-like motif are aided by HSP70 chaperones and transported to lysosomes via the LAMP2A translocase 15. Subsequently, the substrate proteins undergo rapid degradation by various lysosomal hydrolytic enzymes into individual amino acids. The critical role of LAMP2A in CMA is to serve as a key receptor or translocase on the lysosomal membrane. LAMP2A facilitates the recognition and binding of substrate proteins with the KFERQ-like motif, which are chaperoned by heat shock protein family A member 8 (HSPA8). LAMP2A plays a crucial role in translocating these substrate proteins across the lysosomal membrane, enabling their entry into the lysosomal lumen for degradation, and has been reported to be elevated in numerous human cancer tissues 16-19. Meanwhile, the downregulation of CMA reduces lung and gastric cancer cell proliferation. However, limited information exists regarding the role of autophagy in HNSCC.

This study validated STING and TBK1 as novel CMA substrates and provided evidence for CMA-mediated control of their protein levels. Consistent with the enhanced antitumor immune response and tumor growth inhibition attributed to STING and TBK1, we discovered that inhibiting CMA in a STING and TBK1-dependent manner enhanced antitumor immunity 20, 21. Utilizing CMA inhibitors to treat tumor-bearing mice exhibited heightened immune cell infiltration, particularly of effector CD8+ T cells, and a simultaneous augmentation in responsiveness to immune checkpoint blockade (ICB). Finally, our study underscored the significance of targeting LAMP2A in HNSCC to enhance the therapeutic efficacy of PD-1 treatment for patients suffering from HNSCC. Our research enhanced the current understanding of the mechanisms regulating the expression of proteins associated with antitumor immune responses, laying the foundation for the development of novel and specific strategies for therapeutic purposes.

## Results

### STING and TBK1 orchestrate degradation through a lysosomal mechanism independent of macroautophagy

**Figure 1:**
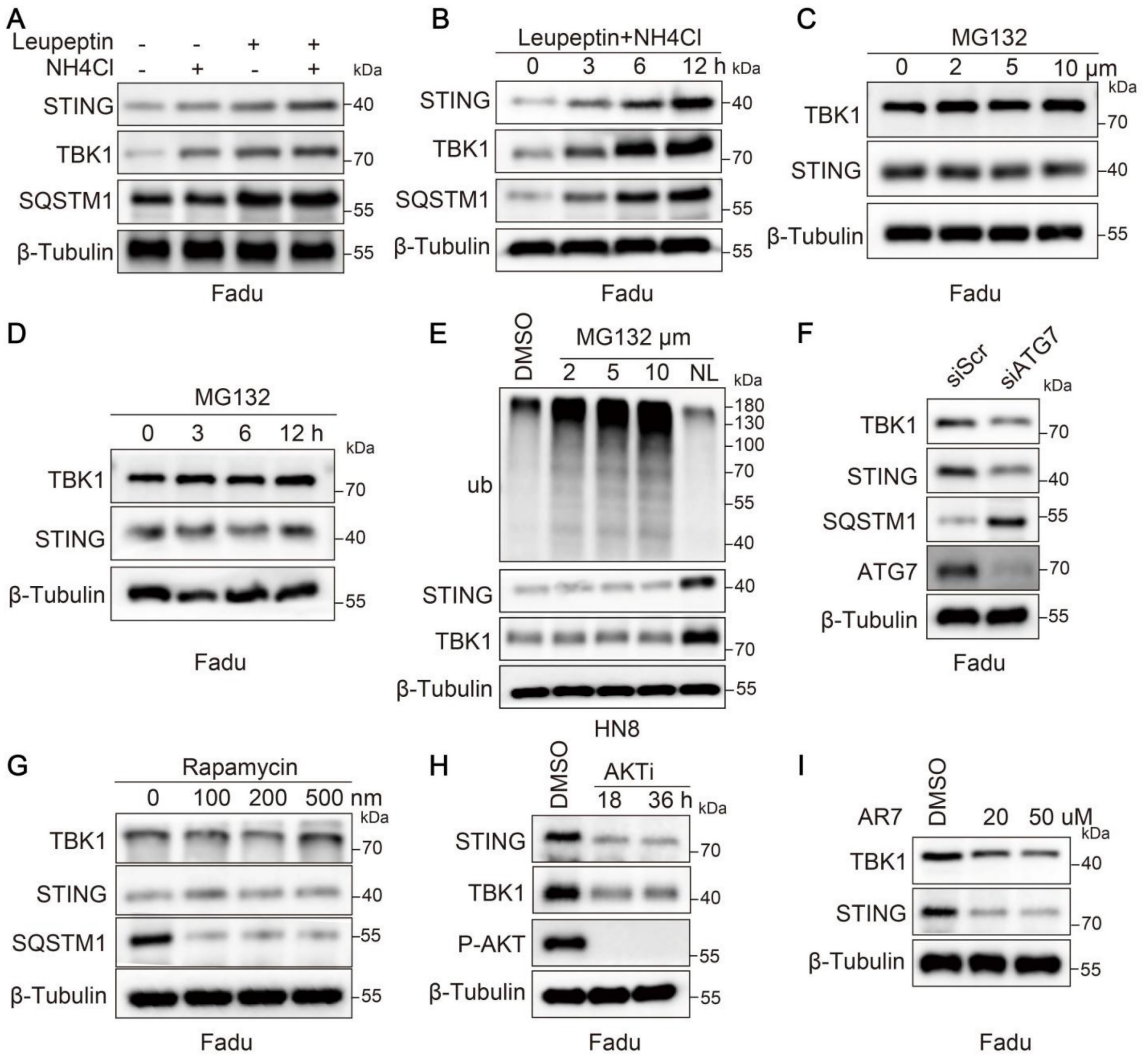
**STING and TBK1 undergo degradation via the lysosomal pathway. (A, B)** Treatment of Fadu cells for 6 h with the lysosomal inhibitor(s) NH4Cl (20 mM) and leupeptin (50 μM) (A) or with a combination of the two for the indicated time (B) leads to the accumulation of STING and TBK1 in Fadu cells. SQSTM1 is shown as a positive control.** (C, D)** Treatment of Fadu cells for 6 h with the indicated concentration of the proteasome inhibitor MG132 (C) or for the indicated time with 5 μM MG132 (D) has moderate to no effects on STING and TBK1 levels. **(E)** Western blot analysis of ubiquitin-conjugated proteins, STING and TBK1 in HN8 cells treated for 12 h either with the indicated concentrations of MG132 or with a combination of 50 μM leupeptin and 20 mM NH4Cl (NL: NH4Cl and leupeptin). **(F)** Silencing autophagy related 7(ATG7) does not cause the accumulation of STING and TBK1 in Fadu cells. SQSTM1 is shown as a positive control. **(G)** Treatment of Fadu cells for 24 h with the indicated concentrations of the macroautophagy-inducing drug rapamycin fails to reduce the levels of STING and TBK1 in Fadu cells. SQSTM1 is shown as a positive control. **(H)** Treatment of Fadu cells with 10 μM of AKT inhibitor decreases STING and TBK1 levels. **(I)** Treatment of Fadu for 24 h with the indicated concentrations of the atypical RARA receptor antagonist AR7 decreases STING and TBK1 levels.

To investigate the degradation mechanism of STING and TBK1 in tumors, we treated the Fadu cell line with proteasome or lysosome inhibitors. Compared to inhibiting the proteasomal degradation pathway, inhibiting lysosomal activity resulted in a more pronounced accumulation of STING and TBK1 (Figure 1A-D and Figure S1A-D). We obtained analogous results in the HN8 cell line (Figure 1E). Further, we validated the drug efficacy by treating Fadu cells with MG132 for 6 hours and assessing ubiquitin-conjugated proteins (Ub-conj) and the apoptotic marker PARP1 (Figure S1E). We ascertained whether STING and TBK1 were degraded via macroautophagy, suppressed by inhibiting ATG5 or ATG7 or stimulated by rapamycin. Inhibition of macroautophagy was notably reflected in the accumulation of SQSTM1/p62; however, it did not elevate the expression levels of STING and TBK1 (Figure 1F-G and Figure S1F). In particular, inhibiting macroautophagy paradoxically increased the degradation of STING (Figure 1F and Figure S1F). This phenomenon may be explained by crosstalk between macroautophagy and other degradation pathways, such as CMA 22-24. LAMP2A, a pivotal lysosome receptor, acts as a CMA accelerator. As macroautophagy was inhibited or activated, LAMP2A was correspondingly activated or suppressed (Figure S1G-H). Silencing LAMP2A resulted in a modest decrease in SQSTM1 and an increase in LC3-II, consistent with an increase in autophagic flux (Figure S1I). CMA was activated when cells were under prolonged starvation conditions, leading to a decline in the levels of STING and TBK1 (Figure S1J). Likewise, AKTi-1/2 and AR7, two chemical activators of CMA, also induced the degradation of STING and TBK1 (Figure 1H-I and Figure S1K-L). The data above collectively indicated that in HNSCC cells, the degradation of STING and TBK1 occurs through a lysosomal mechanism independent of macroautophagy.

### CMA regulates the degradation of STING and TBK1

**Figure 2:**
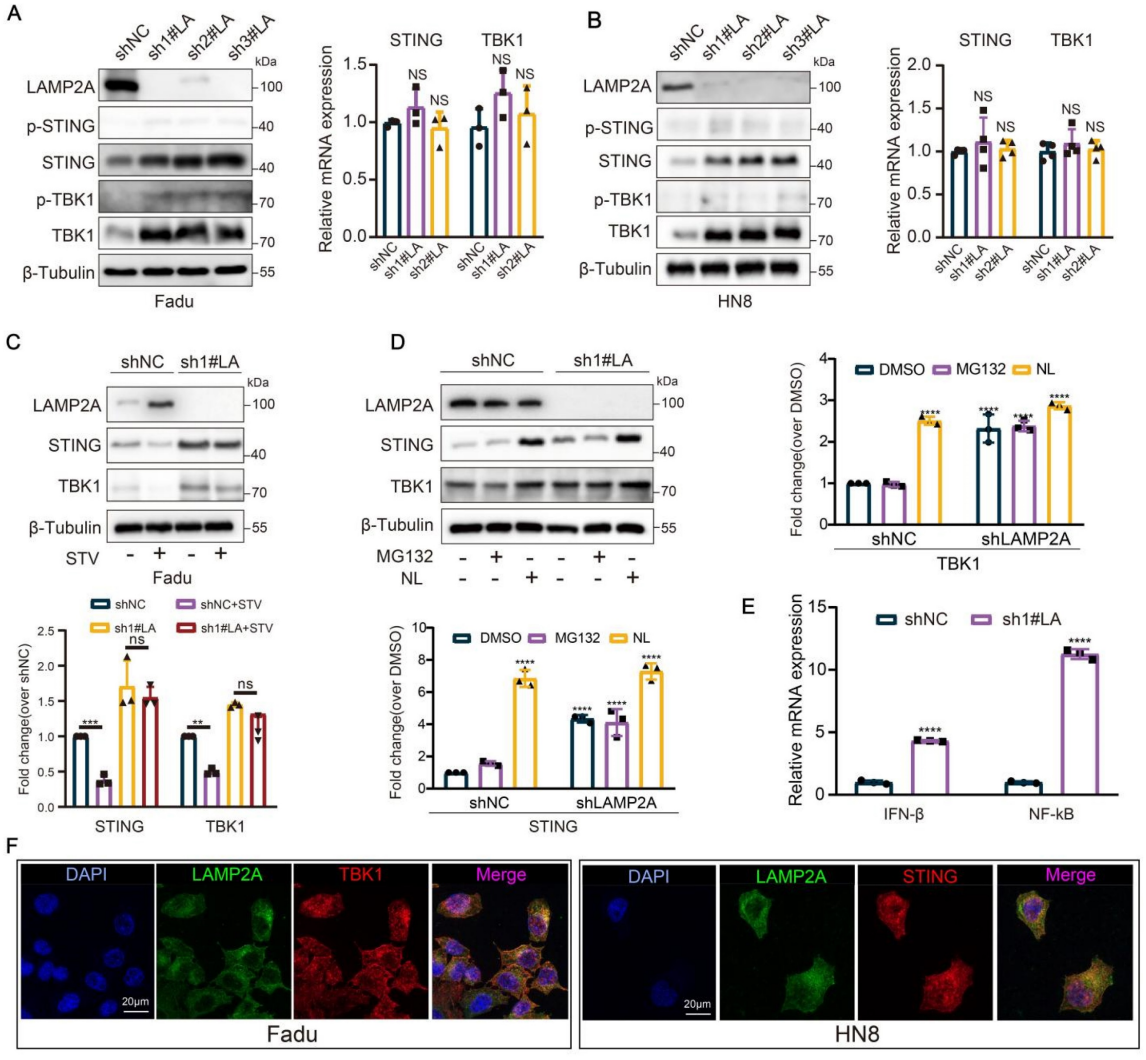
**LAMP2A regulates the degradation of STING and TBK1. (A, B)** Knockdown of LAMP2A causes the protein accumulation of STING, p-STING, TBK1 and p-TBK1 in Fadu and HN8 cells, but thees mRNA levels were not change. **(C)** The knockdown of LAMP2A prevents the degradation of STING and TBK1 induced by starvation. Cells were cultured without serum for 24 hours. **(D)** The expression levels of STING and TBK1 were assessed in shNC or shLAMP2A Fadu cells and treated for 6 hours with 5 uM MG132 or lysosomal inhibitors (NL: 50 uM leupeptin and 20 mM NH4Cl). **(E)** The expression of both STING WT and STING K228R was examined in Fadu cells treated for 6 hours with either 5 uM MG132 or lysosomal inhibitors (NL; 50 uM Leupeptin and 20 mM NH4Cl). **(F)** knockdown of LAMP2A cause an increase in the mRNA levels of IFNβ (a STING target gene) and NF-kB (a common target gene in the STING-TBK1 pathway) in Fadu cells. **(G)** Immunofluorescence reveals the co-localization of the lysosomal marker LAMP2 with STING and TBK1.Means ± SD. NS, not significant, *p < 0.05, **p < 0.01, ***p < 0.001 vs. control.

Like the cellular "stomach," the lysosome serves as the precinct for digesting proteins via CMA. The silencing of the CMA accelerator, LAMP2A, precipitates the accumulation of STING and TBK1 proteins within HNSCC cells (based on LAMP2A expression levels in squamous cell carcinoma of the head and neck cell lines, Fadu and HN8 cell lines were selected for this study), while mRNA expression remains insignificantly altered. However, in the absence of STING agonist, there was no significant change in the protein expression levels of p-STING and p-TBK1 (Figure 2A-B, and Figure S1M-O). Furthermore, silencing of LAMP2A could also rescue the depletion of STING and TBK1 proteins induced by starvation (Figure 2C). To further dissect the role of CMA in the degradation of STING and TBK1, we treated LAMP2A-silenced cells with lysosomal and proteasomal inhibitors separately. Lysosomal inhibition further elevated TBK1 and STING levels; however, proteasomal inhibition did not induce significant alterations in the expression levels of these two proteins (Figure 2D). These observations further underscored that the lysosomal mechanism of CMA is the principal mode of degradation for both proteins. Previous studies have reported that ubiquitination at the STING K288 site is essential for regulating STING degradation and signal termination 25. Therefore, we designed an overexpression plasmid for STING-K288R. Our results showed that lysosomal inhibition also enhanced the expression of the STING K288R mutant (Figure S1P). Similarly, LAMP2A knockdown increased STING K288R mutant expression (Figure S1Q), while ATG7 silencing did not produce a similar effect (Figure S1R), further confirming that CMA controls STING expression. Meanwhile, LAMP2A increased the mRNA levels of the downstream target genes IFNβ and NF-kB of STING-TBK1 (Figure 2E). Furthermore, in Fadu cells, we observed a robust colocalization of STING and TBK1 with the lysosomal marker LAMP2A (Figure 2F). In conclusion, CMA is the primary modality for regulating STING and TBK1.

### STING and TBK1 serve as substrates for CMA

**Figure 3:**
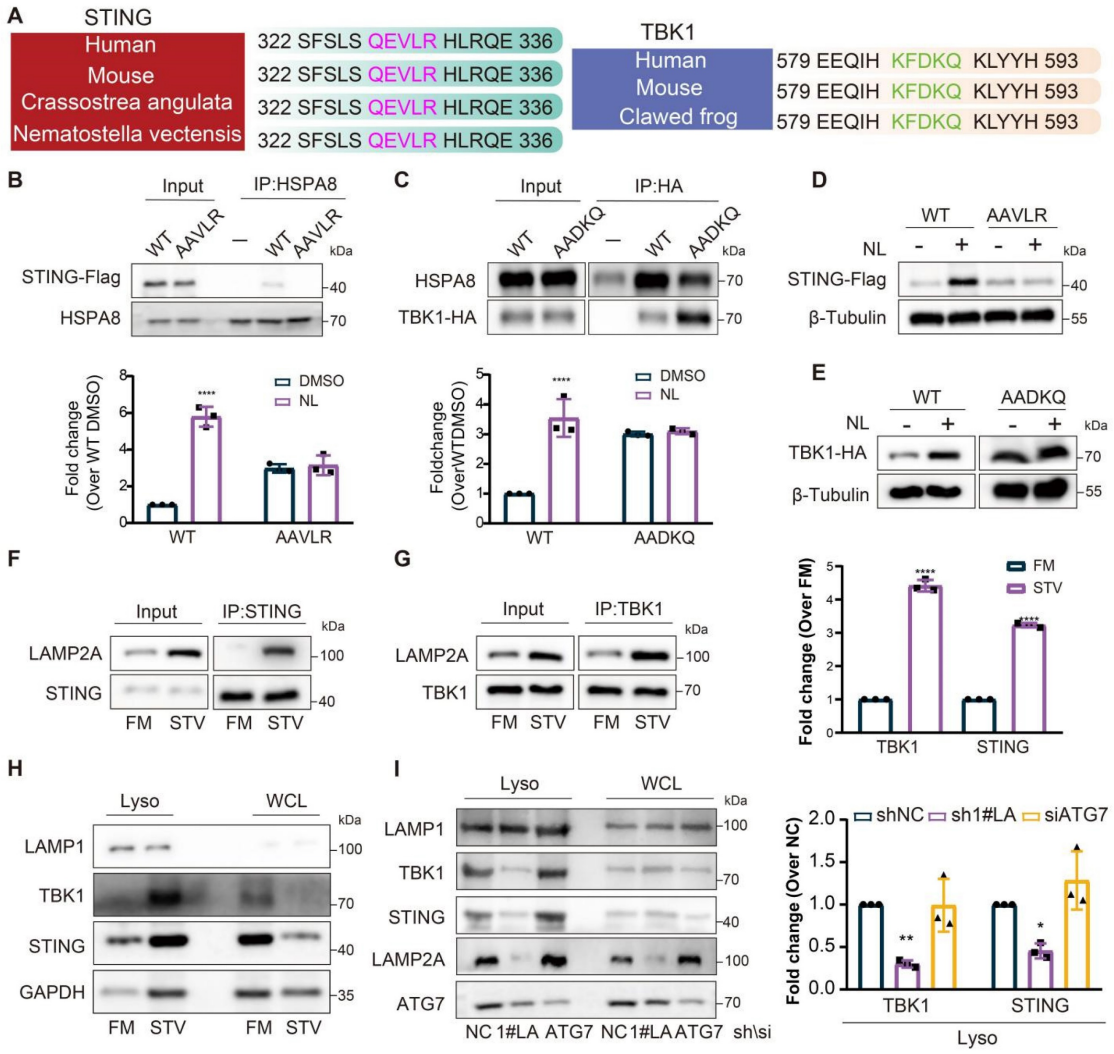
**STING and TBK1 as new substrates of CMA. (A)** The putative KFERQ motif is depicted in the sequences of STING and TBK1 across multiple species. **(B)** FLAG-STING WT, but not FLAG STING AAVLR, is detectable in HSPA8 immunoprecipitates. **(C)** More HSPA8 is detectable in HA-TBK1 WT than in TBK1 AADKQ immunoprecipitates. **(D, E)** Mutation of the KFERQ motif elevates the expression of STING(D) and TBK1(E) and renders them unresponsive to treatment with lysosomal inhibitors (NL; 20 mM NH4Cl and 50 uM leupeptin, 6 h). **(F, G)** Starvation induces stronger interaction of between STING (F) or TBK1 (G) and LAMP2A. **(H)** Starvation promotes the accumulation of STING and TBK1, and the canonical CMA substrate GAPDH in lysosomes isolated from Fadu cells. Cells were cultured without serum for 24 h. **(I)** Loss of LAMP2A, but not ATG7, reduces starvation-induced lysosomal localization of STING and TBK1. Means ± SD. NS, not significant, *p < 0.05, **p < 0.01 vs. control.

The results above indicated that knockdown of LAMP2A could inhibit the degradation of STING and TBK1, while activation of CMA enhanced it. Subsequently, we explored the potential of STING and TBK1 to serve as substrates for CMA. To be a CMA substrate, a protein must contain a KFERQ-like motif, recognized by HSPA8, which is essential for substrate lysosomal translocation. By comparing the amino acid sequences of STING and TBK1 across different species, we identified the presence of a KFERQ-like motif (Figure 3A). Mutations in the KFERQ-like motif reduced the interaction between STING and TBK1 with HSPA8 (Figure 3B-C). Moreover, when cells with mutated KFERQ-like motifs were treated with lysosomal activity inhibitors, the accumulation of STING and TBK1 was attenuated (Figure 3D-E). Starvation of cells increased STING and TBK1 accumulation within lysosomes and promoted the interaction of STING and TBK1 with LAMP2A (Figure 3F-H), providing further evidence that CMA is the primary lysosomal degradation mechanism for STING and TBK1. Silencing LAMP2A instead of ATG7 inhibited the degradation of starvation-induced STING and TBK1, ruling out macroautophagy's involvement in the degradation of these two proteins (Figure 3I). These data also suggested that STING and TBK1 are novel substrates for CMA.

### LAMP2A deficiency promotes type I IFN signaling

**Figure 4:**
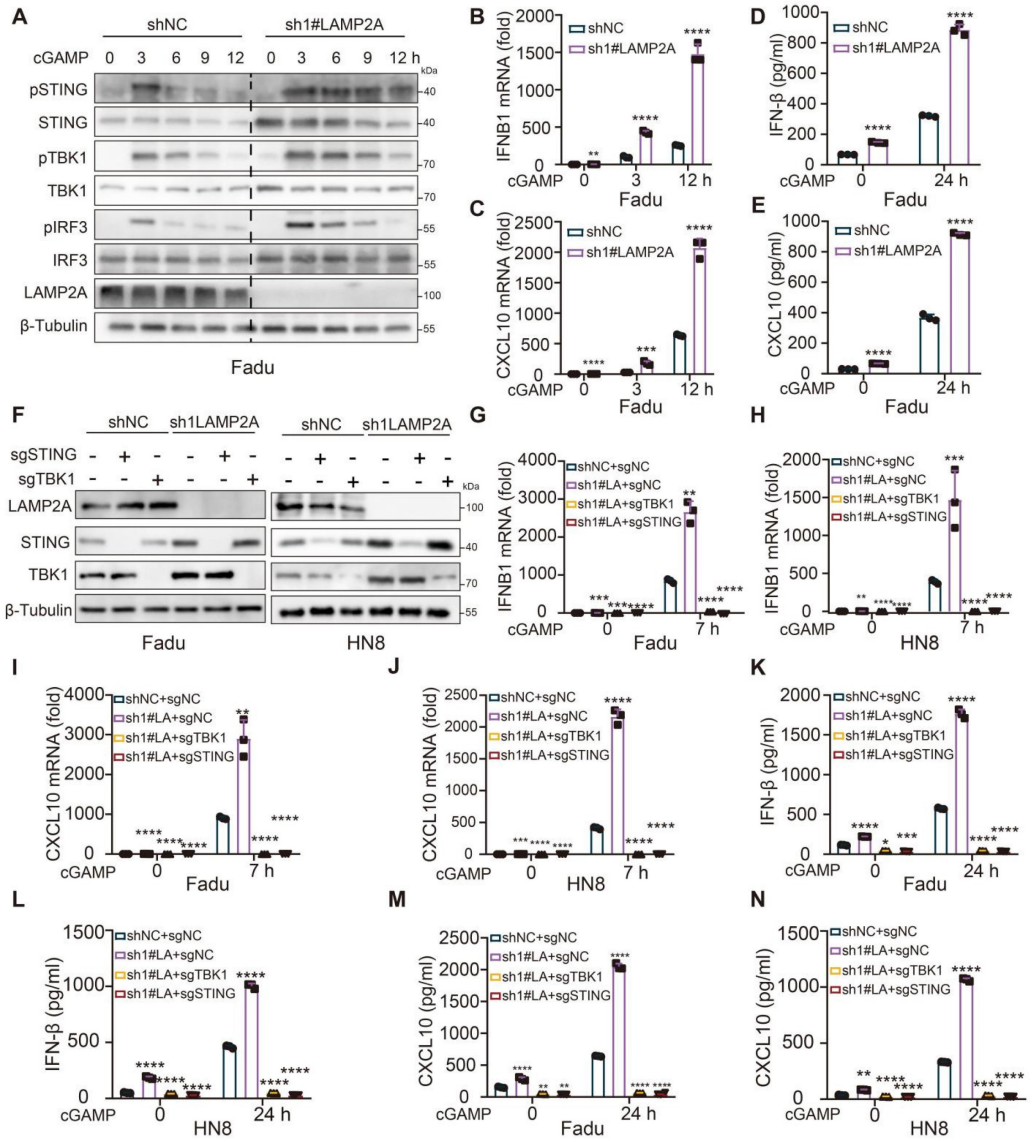
**LAMP2A deficiency chronically activates STING-mediated type I IFN signalling. (A)** Immunoblot (IB) analysis of the STING and TBK1 signaling cascade. ShNC and shLAMP2A cells were stimulated with the cGAMP (10 μg/mL) for 0, 1, 3, 5 or 7 h. The total and phosphorylated proteins immunoblotted are identified on the left. **(B, C)** Quantitative polymerase chain reaction (PCR) analysis of IFNB1 (B) and CXCL10 (C) mRNA in Fadu treated with the indicated treatment. **(D, E)** Enzyme-linked immunosorbent assay (ELISA) analysis of IFN-β (D) and CXCL10 (E) in Fadu treated with the indicated treatment. **(F)** IB analysis was validate the knockout efficiency of STING and TBK1. **(G-K)** qPCR analysis of IFNB1(G-H) and CXCL10(J-K) expressio for the indicated times after stimulated with cGAMP (10μg/ml) and ELISA. **(L-O)** analysis of IFN-β (L-M) and CXCL10 (N-O) production in the cell culture supernatants. Data are representative of three independent experiments with similar results means ± SD. NS, not significant, *P < 0.05, **P < 0.01, and ***P < 0.001, ****P < 0.0001.

Since STING and TBK1 are essential kinases involved in virus-triggered type I IFN signaling, we tested whether LAMP2A regulated the STING and TBK1-dependent antiviral response. After stimulation with the endogenous STING ligand cGAMP or STING agonist DMXAA, we observed more substantial and prolonged activation of pSTING, pTBK1, and pIRF3 in shLAMP2A compared to shNC cells (Figure 4A, Figure S2A and Figure S2F). As anticipated, qPCR showed that LAMP2A knockdown enhanced cGAMP or DMXAA-induced IFNB1 and CXCL10 expression in HNSCC cells (Figure 4B-C and Figure S2B-C). Similar results were obtained using ELISA (Figure 4D-E, and Figure S2D-E). However, FNB1 and CXCL10 expression was abolished by STING or TBK1 deletion (Figure 4F-N and Figure S2F-J). Analogous conclusions were derived under treatment with diclofenac (CMA inhibitor) (Figure S2K-O). These results demonstrated that LAMP2A knockdown enhances IFNB1 production and the cellular antiviral response by inhibiting the CMA-mediated degradation of STING and TBK1.

### Absence of LAMP2A enhances antitumor immunity in a STING- and TBK1-dependent manner and reduces HNSCC growth

**Figure 5:**
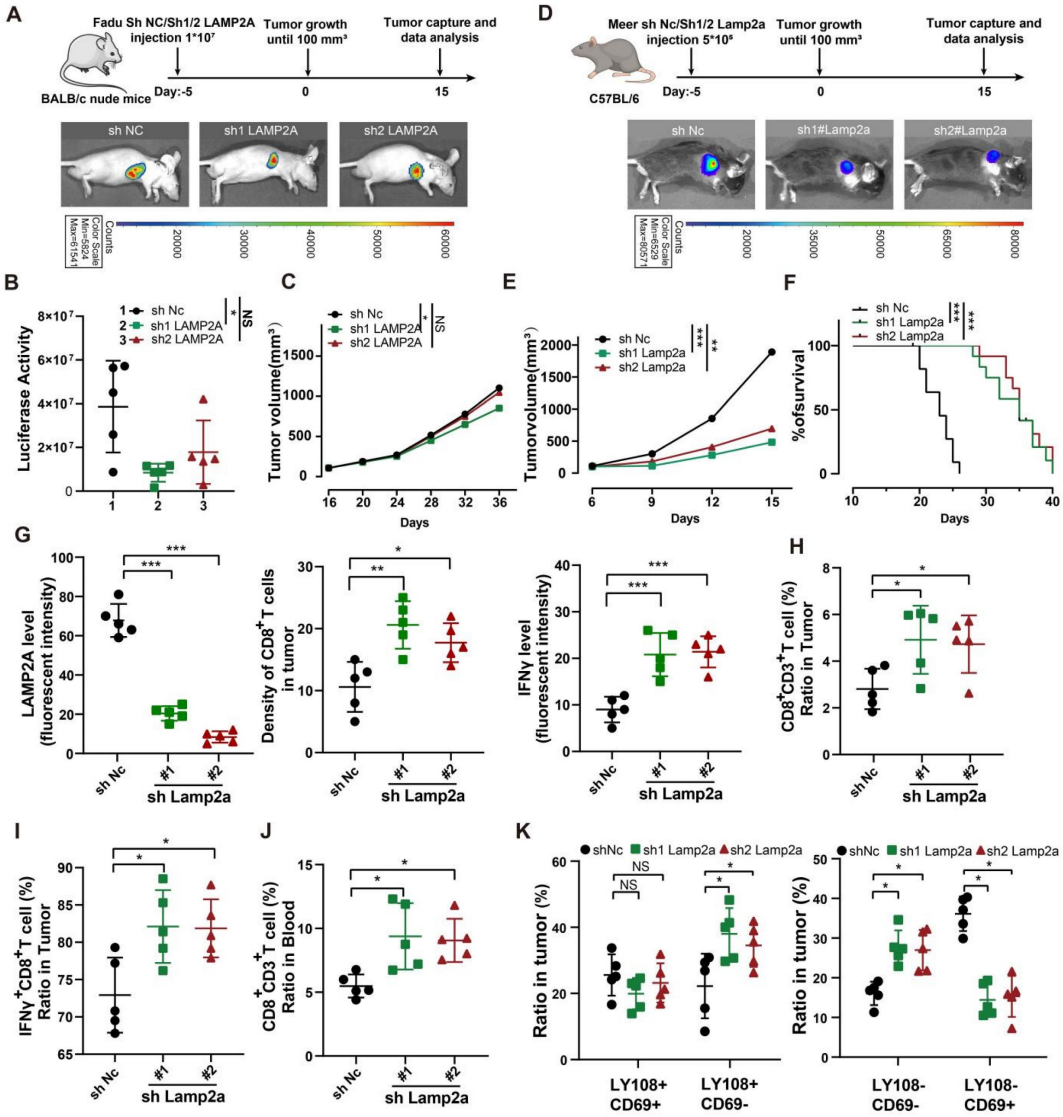
**In immune-competent mouse models, inhibition of Lamp2a induces antitumor immunity, thereby attenuating tumor growth. (A)** A schematic view of the treatment plan and BLAB/C nude mice were inoculated with GFP-Luc-shNC, or GFP-Luc-shLAMP2A Fadu cells. **(B, C)** Summary of luciferase activity(B) and volume data(C) of Fadu tumors harvested after euthanizing the mice.** (D)** A schematic view of the treatment plan and subcutaneous tumor images of the Meer cells. **(E)** Summary of volume data of Meer tumors harvested after euthanizing the mice.** (F)** Kaplan-Meier survival curves for each group. **(G)** mIHC Quantitative shLamp2a or shNC Meer xenograft GZMB, Lamp2a and CD8. **(H, I)** FACS of CD3+ in CD45+ cells and IFNγ+ in CD8+ cells TILs from shLamp2a or shnc Meer xenografts quantification. **(J)** FACS of CD8+ in CD3+ cells from blood of shLamp2a or shnc Meer xenografts quantification. **(K)** FACS of LY108(-/+) CD69(-/+) cells in CD3+ TILs from shLamp2a or shNC Meer xenografts and quantification. mIHC multiple immunohistochemical , *p < 0.05, **p < 0.01, ***p < 0.001 vs. control.

STING and TBK1 are pivotal molecules that initiate antitumor immunity 26. We conducted *in vivo* experiments to explore whether LAMP2A silencing contributes to an anti-cancer phenotype. We observed that LAMP2A depletion slightly inhibited the growth of tumors in nude mice with no significant differences in survival between groups (Figure 5A-C, Figure S3A-B). Furthermore, we implanted LAMP2A-deficient Meer cells subcutaneously in C57BL/6 mice, compared them to immunodeficient nude mice, and found the difference in tumor growth to be more pronounced in immunocompetent mice (Figure 5D). This observation suggested that the absence of LAMP2A stimulates a more robust antitumor immune response in the context of a complete immune system. Compared to the control group, the expression of STING and TBK1 was higher (Figure S3C), and the tumor volume in the LAMP2A-deficient group was smaller with longer survival times, which was consistent with the results from *in vitro* experiments (Figure 5E, F).

Our findings indicated that blocking a pro-cancer protein can upregulate two critical anti-cancer proteins. STING is pivotal in the innate immune response triggered by viral, bacterial, and parasitic infections, the body's antitumor immune processes, and cellular autophagy, prompting us to compare the infiltration of immune cells in tumor tissues in the absence of LAMP2A (Figure S3D). Immunohistochemical and flow cytometry results revealed a significant increase in the infiltration density and activity (IFNγ+ and IFNγ+PD1+) of CD8+ T cells due to the loss of LAMP2A (Figure 5G-J Figure S3E-L). However, there was no difference in the number of PD-1+CD8+ T cells in peripheral blood among the three groups (Figure S3J and Figure S3M).

Further, we classified the exhausted T cells into four stages based on Ly108 and CD69. In the LAMP2A-deficient group, the number of cells representing the cytotoxic effector stages, TexProg2 and TexInt, was significantly higher than in the control group. In contrast, the number of cells representing the quiescent stages, TexProg1 and TexTerm, was markedly reduced (Figure 5K and Figure S3N), substantiating the ability of LAMP2A to induce an antitumor immune response. We demonstrated that LAMP2A deficiency exerted antitumor immunity mediated by STING-TBK1 by treating C57BL/6 mice inoculated with LAMP2A-deficient Meer cells with STING (SN-011) and TBK1 (GSK8621) inhibitors (Figure S4A). We found that both SN-011 and GSK8621 partially reversed LAMP2A-induced tumor shrinkage (Figures S4B-C).

Based on immune infiltration assessment using the TCGA dataset, we found that LAMP2A negatively correlated with CD8+ T and NK cells. In contrast, it positively correlated with myeloid-derived suppressor and regulatory T cells (Figures S4D- E). Moreover, compared to the high-expression patients, the low-expression subgroup exhibited significantly greater TCR richness (Figure S4F) and higher quantities of CD8, plasma cells, activated memory CD4 T cells, and neutrophils. Conversely, the high-risk patients demonstrated a higher abundance of resting CD4 cells and M2 macrophages (Figure S4G). In conclusion, silencing LAMP2A can enhance antitumor immunity dependent on STING and TBK1 and suppress HNSCC growth.

### Absence of LAMP2A exhibits a synergistic effect with PD-1 monoclonal antibody treatment *in vivo*

**Figure 6:**
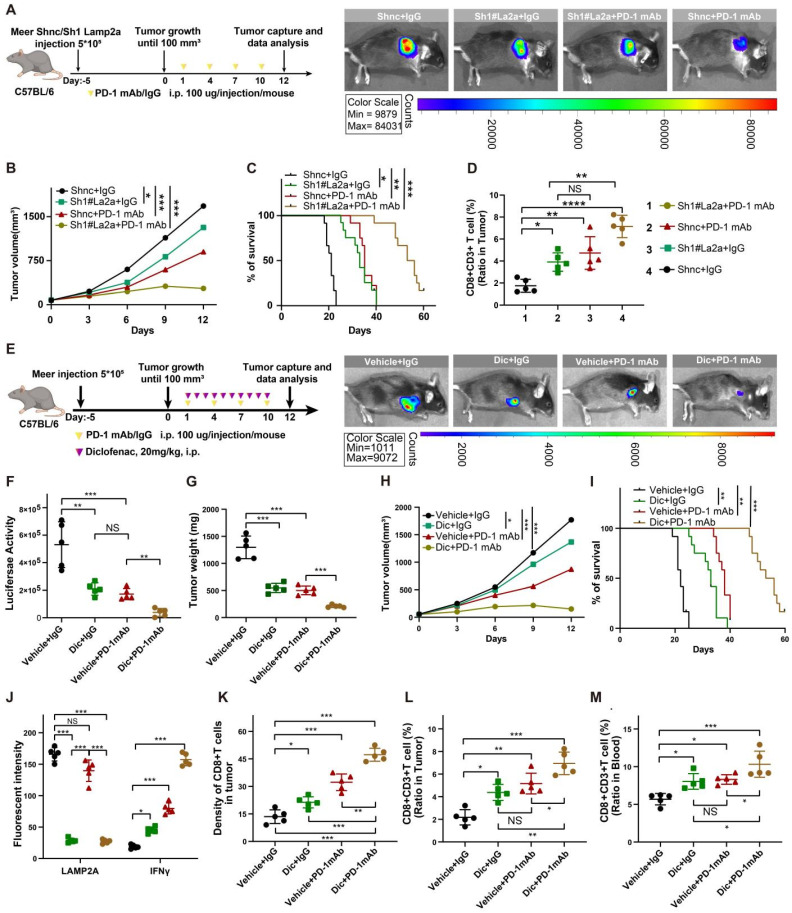
**Improved *In vivo* Anti-tumor Effect of PD-1 Blockade and shLamp2a Cotreatment. (A)** A schematic view of the treatment plan and C57BL/6 mice received GFP-Luc shnc, or sh1Lamp2a Meer cells were injected with luciferin. **(B)** Summary of volume data of Meer tumors harvested after euthanizing the mice. **(C)** Kaplan-Meier survival curves for each group. **(D)** FACS of CD8+ in CD3+ TILs from different groups Meer xenografts and quant ification. **(E)** A schematic view of the treatment plan and different groups of C57BL/6 mice received GFP-Luc Meer cells were injected with luciferin. **(F-I)** Summary of luciferase activity(F), tumor weight(G), volume data(H) and Kaplan-Meier survival curves(I) for each group. **(J, K)** mIHC quantification of IFNγ, Lamp2a, and CD8(K) expression in transplanted tumors. **(L, M)** FACS of CD8+ CD3+ TILs from from heterologous Meer mouse transplant tumors (L) and blood samples (M) in different groups. log-rank test for survival comparison. Means ± SD. NS, not significant, *p < 0.05, **p < 0.01, ***p < 0.001 vs. control.

Since the loss of LAMP2A recruited more cytotoxic CD8+ T cells, we next investigated whether manipulating LAMP2A could enhance the efficacy of ICB therapy. We treated immunocompetent mice bearing tumors of Sh1#Lamp2a or ShNc knockout Meer cells with PD-1 monoclonal antibody (PD-1 mAb) (Figure 6A). The tumor volume in the Sh1#Lamp2a combined with the PD-1 mAb group was smaller, and the survival time was longer than that of the other groups (Figure 6B-C). As expected, the Sh1#Lamp2a and PD-1 mAb combination also recruited more CD8+ T cells (Figure 6D and Figure S5A).

The active involvement of non-steroidal anti-inflammatory drugs (NSAIDs) in CMA has been reported with studies affirming that NSAIDs, including diclofenac, augment the degradation of LAMP2A and inhibit CMA activity 27, 28. *In vitro* experiments confirmed a significant decrease in the expression levels of LAMP2A with an escalating concentration gradient and prolonged exposure to diclofenac (Figure S6B). We administered diclofenac and PD-1, separately and in combination, to murine models with HNSCC tumor xenografts (Figure 6E). Compared to the control group, diclofenac treatment significantly reduced LAMP2A expression and markedly inhibited the growth of MEER tumors. As expected, the combination treatment of diclofenac and PD-1 monoclonal antibody demonstrated the most potent antitumor effect compared to individual treatments (Figure 6F-I).

We also examined changes in immune markers in tumor tissues and blood from different treatment groups. In the combination treatment group, CD8+ T cell infiltration and IFNγ expression were highest, while the expression of LAMP2A was relatively low (Figure 6J-M and Figure S5C-F). However, there were no significant differences in PD-1+CD8+ T cells among various groups (Figure S5G). Our data suggested that LAMP2A is a potential therapeutic target for HNSCC, and diclofenac has a synergistic antitumor effect with PD-1 monoclonal antibody.

### LAMP2A is associated with the therapeutic efficacy of PD-1 monoclonal antibody treatment in HNSCC patients

To demonstrate the oncogenic functionality of LAMP2A, we conducted immunoblot analysis on cancer and adjacent tissues from surgically treated HNSCC patients. The results indicated significant upregulation of LAMP2A in cancer tissues compared to adjacent normal tissues, accompanied by decreased expression of STING and TBK1 (Figure S6A), which was in line with our findings from *in vivo* experiments (Figure 5-6). Immunohistochemical analysis from public databases and clinical samples further confirmed the relatively high expression of LAMP2A in cancer tissues (Figure S6B-C). Also, univariate COX regression analysis revealed that patients with high LAMP2A expression were more likely to have poorly differentiated tumors, high KI67 expression, lymph node-positive metastasis, and advanced T stage (P<0.05) (Table S1).

**Figure 7:**
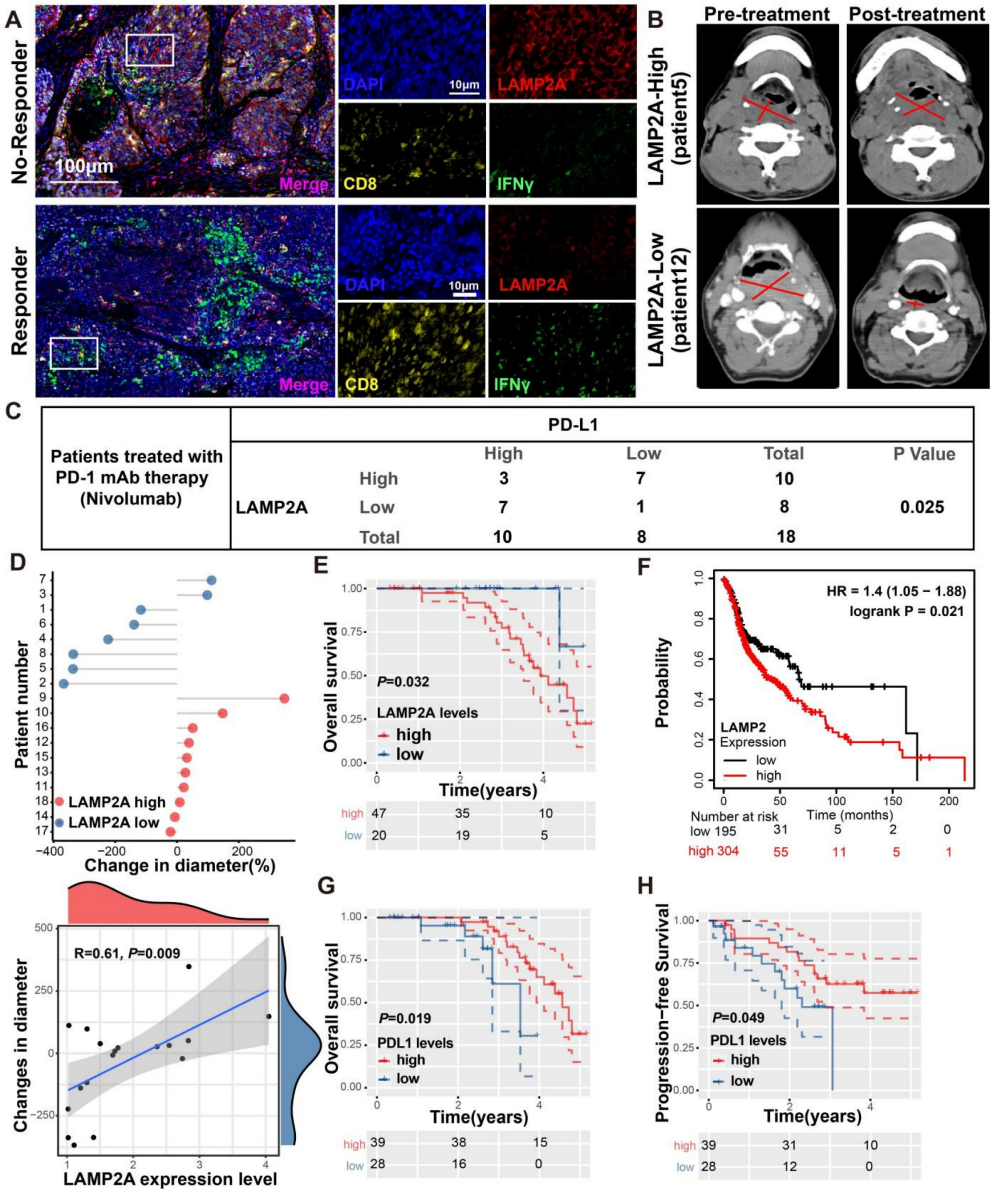
**LAMP2A exhibits a negative correlation with PD-L1 expression in patient samples from HNSCC. (A, B)** Two HNSCC patients who underwent PD-1 monoclonal antibody (Nivolumab) therapy, one showing a positive response (Pt.12) and the other a non-responsive case (Pt.5), were examined. mIHC was performed using antibodies against LAMP2A, CD8 and IFNγ in the tumor tissues(A). Tumor diameter, as indicated by a red line on CT imaging(B), was assessed by the radiologist. **(C)** The expression of LAMP2A is inversely correlated with the expression levels of PD-L1. **(D)** The difference in tumor diameter for all patients, where those with increased tumor diameter are colored by red(upper). Quantitative correlation between the change in tumor diameter and LAMP2A expression levels(lower). **(E, F)** Differentially expressed LAMP2A HNSCC patients OS analysis in Harbin medical university affiliated tumor hospital cohort(E) and TCGA cohort(F). **(G, H)** Differentially expressed PDL1 HNSCC patients OS(G) and PFS(H) analysis in Harbin medical university affiliated tumor hospital cohort. log-rank test for survival comparison.

We performed multiplex immunohistochemistry (mIHC) to assess the protein expression levels of LAMP2A, CD8, and IFNγ in biopsies from HNSCC who received PD-1 monoclonal antibody treatment. Among the 18 patients recruited, 11 exhibited a positive response to PD-1 monoclonal antibody treatment and were categorized as responders, while the remaining 7 patients did not respond well and were classified as non-responders. Compared to patients with high LAMP2A expression, those with low LAMP2A expression showed significantly increased tumor-infiltrating CD8+T cells and IFNγ expression, indicating a favorable response to PD-1 monoclonal antibody treatment (Figures 7A-B). Our mIHC results revealed a negative correlation between LAMP2A and PD-L1 expression in 14 out of 18 patients (Figure 7C; p=0.025), suggesting that LAMP2A could predict PD-1 monoclonal antibody treatment efficacy.

Figure 7D displays the differences in tumor diameter among all patients. We observed a significant positive correlation between the relative increase in tumor diameter and LAMP2A expression levels. Subsequently, we assessed the correlation of LAMP2A and PD-L1 with the prognosis of HNSCC patients using our data and the TCGA public database. As shown in Figure 7E-H, patients with low LAMP2A and high PD-L1 expression had longer progression-free survival and overall survival. These patients may benefit from LAMP2A antagonists, which inhibit tumor proliferation and enhance the efficacy of PD-1 monoclonal antibody treatment by upregulating tumor PD-L1 levels, thus contributing to the synergistic improvement of immunotherapy efficacy.

## Discussion

CMA plays a crucial role in regulating the tumor immune microenvironment, maintaining tumor cell stemness, and reprogramming tumor metabolism 17, 29-31. The STING-TBK1 pathway is pivotal for activating cytotoxic T cells vital for tumor immune surveillance and successful immunotherapy. In many cancer patients, this pathway is often downregulated. The exact mechanisms underlying the silencing of STING and TBK1 in tumor patients remain unclear. LAMP2A is a critical "booster" for the CMA lysosomal degradation mechanism. Dysregulation of CMA is implicated in numerous human diseases, especially cancer, highlighting LAMP2A as a potential target for cancer therapy. It is of note that LAMP2 generates three variants through alternative splicing: LAMP2A, LAMP-2B, and LAMP-2C 32. The oncogenic role of LAMP2A has been confirmed in various cancers. LAMP2A can promote the malignant behavior of several cancers, including hepatocellular carcinoma, prostate cancer, lung cancer, glioblastoma, and breast cancer, among others 33, 34. However, there is limited information on the crucial role of LAMP2A in the tumor microenvironment, and the impact of targeting LAMP2A on enhancing antitumor immunity has not been reported.

In this study, we have demonstrated the pivotal role of LAMP2A in the progression of HNSCC and its implications for immunotherapy through three key aspects: cell experiments, clinical samples from our center, and external data. We have provided evidence that CMA controls STING and TBK1 levels, thereby expanding the range of cancer-related proteins regulated by CMA. The silencing of LAMP2A, a translocase enzyme within CMA, increases the levels of both proteins without altering mRNA expression. Further analysis has revealed that STING and TBK1 are direct substrates of CMA, uncovering a novel mechanism for controlling the expression of these two cancer-related proteins. LAMP2A knockdown enhances IFNB production and the cellular antiviral response by inhibiting the CMA-mediated degradation of STING and TBK1. AP-1 is a key protein that regulates the phosphorylation of STING, encapsulating it in transport vesicles for delivery to the endolysosomal system, where STING is degraded 35. LAMP2A may control this critical lysosomal degradation process. The study by Xibao *et al.* is consistent with our findings, suggesting that TBK1 contains a typical CMA motif and undergoes degradation via CMA. In macrophages, the deletion of USP19 following viral infection leads to an increase in TBK1 and activation of type I interferon signaling 36. These studies highlight the lysosome as a key site for the degradation of STING and TBK1. Given these discoveries, we postulate that LAMP2A may represent a potential therapeutic target for HNSCC patients.

It is well known that membrane proteins such as STING primarily enter the lysosomal lumen through three main pathways: (1) macroautophagy, (2) membrane fusion, or (3) direct encapsulation 25. Kuchitsu *et al.* have demonstrated that STING enters the lysosomal lumen through "direct encapsulation" 37. In non-tumor cells, activated STING shows a greater tendency toward lysosomal degradation than its stable form 25. Kuchitsu's study and our findings indicated that even inactive STING undergoes lysosomal degradation. However, the mechanism by which this inactive STING reaches the lysosome remains unclear.

Up to 60% of HNSCC patients are diagnosed at an advanced local stage, and approximately 50 - 60% experience relapse, often without a cure. ICB therapy has shown clinical benefits for HNSCC patients with a high tumor mutation burden and CPS ≥ 1 38. However, these patients represent a minority of those with recurrent disease, and their response rate to ICI therapy is only 16% 39. This may be partly due to the poor tumor infiltration of cytotoxic CD8+ T cells and the accumulation of immunosuppressive MDSCs and Treg cells in the immune microenvironment of HNSCC patients. Therefore, improving the "cold" tumor state is a potential approach to enhance the effectiveness of ICB therapy in advanced HNSCC patients. In this context, our *in vivo* experiments have demonstrated that targeting the CMA translocase enzyme LAMP2A inhibits the degradation of STING and TBK1, enhancing the infiltration of IFNγ+CD8+ T cells and thus improving the "cold" tumor state. Importantly, we emphasize the potential benefits of using a small molecule LAMP2A inhibitor in combination with PD-1 therapy for tumor patients with high LAMP2A and low PDL1 expression.

In summary, we elucidated the mechanism and role of LAMP2A in antitumor immune responses. Targeting LAMP2A inhibits the degradation of STING and TBK1, thereby enhancing IFNB production and cellular antiviral responses. The combination of LAMP2A inhibitors and anti-PD-1 therapy showed an excellent synergistic effect in HNSCC, highlighting the potential of LAMP2A as a therapeutic target for HNSCC.

## Materials and Methods

### Cell culture

DOK (Dysplastic Oral Keratinocyte) and HNSCC (Fadu, Tu212, Tu686, HN8, and Cal27) cell lines, along with murine-derived head and neck tumor cell line Meer and the human embryonic kidney 293T (HEK293T) cell line, were used in this study. Tu686 cell line was kindly provided by Dr. Zhuo Chen (the Cancer Research Center at Emory University, USA), and the mouse HNSCC cell line MEER was generously gifted by Dr. joseph Califano (University of California San Diego, USA). The remaining cell lines were acquired from the ATCC cell repository or the cell repository of the Chinese Academy of Sciences. DOK and Tu212 cells were cultured in 1640 medium (Gibco, USA), Tu686 in DMEM/F12 medium (Gibco, USA), and Meer in a mixed medium consisting of DMEM (Gibco, USA), F12 (Gibco, USA), Fetal Bovine Serum (Gibco, USA), and supplements including hydrocortisone, transferrin, insulin, Tri-iodo-Thyronine, mouse EGF. Other cell lines were cultured in DMEM medium. All these media were supplemented with 10% fetal bovine serum (FBS; Gibco, USA) and 1% Penicillin/Streptomycin (C100C5, NCM Biotech, China). Cell lines were incubated in a humidified chamber with 37 °C in 5% CO2. All experiments were conducted with mycoplasma-free cells.

### Antibodies and regents

The antibodies used for immunoblot and immunoprecipitation were listed as follows: anti-STING (ab252560, Abcam), anti-NAK/TBK1 (ab40676, Abcam), anti-LAMP2A (ab125068, Abcam), Anti-Ubiquitin (ab134953, Abcam), anti-LC3-I/II antibody (ab128025, Abcam), anti-ATG5 (#12994, CST, Boston, USA), anti-ATG7 (#8558, CST), anti-SQSTM1 (#8025, CST), p-AKT (#9271, CST), HSPA8 (10654-1-AP, Proteintech), anti-PARP1 (#9542,CST) and anti-DDDDK tag (#14793, CST), GAPDH (#5174, CST), Tubulin(#5568,CST), HA-Tag (#3724, CST), anti-p-TBK1 (#5483, CST), anti-pSTING (Ser365) (72971, CST), anti-IRF3 (1:1000; 11312-1AP, Proteintech, USA), anti-p-IRF3 (#29047S, CST), HRP-labeled secondary antibody (Anti-rabbit IgG #7074S and Anti-mouse IgG #7076S, CST). The chemicals used were listed as follows: NH4Cl (Ammonium chloride, A9434, Sigma, St. Louis, MO, USA), Leupeptin (103476-89-7, MP Biomedicals, California, USA), MG132 (S2619, Selleck), and CHX (cycloheximide, S7418, Selleck), AKTi-1/2(AKT inhibitor VIII, HY-10355, MCE), AR7 (HY-101106, MCE), Rapamycin(553210, Sigma), D-Luciferin Sodium Salt (40901ES, Yeasen, China), C-176(SML2559, Sigma), GSK8612(SML2721, Sigma), Diclofenac Sodium (D6899, Sigma).

### Plasmids and transfection

Single guide RNAs (sgRNAs) were cloned into lentiCRISPR v2 (Addgene # 52961) and shRNA were cloned into pLKO.1 puro (Addgene #8453) or pLKO.1 hygro(Addgene #24150). siRNAs targeting siLAMP2A, siATG5, siATG7 and negative control (NC) siRNA transfected into cells with a riboFECT™ CP Transfection Kit (RiboBio). Sequences are provided in Supplementary Table S2. The full-length open reading frame of human STING-Flag (NM_198282) and TBK1-HA (NM_013254) was cloned into pLVX-Puro lentivector. All constructs were confirmed by DNA sequencing. For lentivirus production, 5 µg of plasmid DNA, 4 µg of psPAX2 (Addgene #12260), 3 µg of pMD2.G (Addgene #12259), and 36 uL of Lipofectamine 3000 (Thermo Fisher Scientific, L3000015) were mixed and added to HEK293T cells in a 6 cm culture dish. Media was changed 6 hours afte and the supernatant was collected at 48 and 72 hours posttransfection.

### Lysosome enrichment assays

Lysosome enrichment was conducted with a lysosome enrichment kit for tissues and cultured cells (Thermo Fisher Scientific, 89839) following the manufacturer's instructions.

### Quantitative real-time PCR (qRT-PCR)

Total RNA was extracted utilizing the TRIzol reagent, followed by reverse transcription into complementary DNA (cDNA) through employment of the PrimeScript RT reagent kit (TaKaRa, Tokyo, Japan), in strict accordance with the instructions stipulated by the manufacturer. The subsequent quantitative real-time PCR (qRT-PCR) analysis was executed employing the SYBR Green qPCR Master Mix (Takara). The sequences of PCR primers were as follows: Primers used are listed in Supplementary Table S3.

### Opal multiplex immuno-histochemistry

Wax-embedded paraffin sections from formalin-fixed specimens were deparaffinized and rehydrated. Subsequently, endogenous horseradish peroxidase (HRP) was blocked using a 3% hydrogen peroxide solution for 30 minutes. Heat-induced epitope retrieval was performed in a citrate buffer (95 °C, 5 minutes). Following overnight incubation with the primary antibody at 4 °C, fluorescent development was carried out using the Opal 7-Color Automation IHC Kit (Akoya Biosciences, Cat # NEL821001KT) following the manufacturer's instructions. The images were captured using the Vectra® Polaris™ Automated Quantitative Pathology Imaging System and analyzed with inForm Tissue Finder and phenoptr™ software. This study employed formalin-fixed, paraffin-embedded primary head and neck squamous cell carcinoma samples from the Cancer Hospital of Harbin Medical University. The research was carried out in compliance with the approved protocol of the Clinical Research Ethics Committee of the Cancer Hospital of Harbin Medical University, and written informed consent was obtained from all participating patients.

### Immunoblotting and co-immunoprecipitation

Tissue specimens were rapidly frozen in liquid nitrogen, pulverized utilizing a mortar and pestle, and subsequently lysed in T-PER Tissue Protein Extraction Reagent (Thermo Fisher Scientific, #78510) with Halt Protease and Phosphatase Inhibitor Cocktail (Thermo Fisher Scientific, #78445). Protein concentrations were assessed employing the Pierce BCA Protein Assay Kit (Thermo Fisher Scientific). Proteins were separated with 8%-12% sodium dodecyl sulfatepolyacrylamide gel electrophoresis (SDS-PAGE) and transferred to the nitrocellulose membrane. After blocking, the membranes were incubated in primary antibodies at 4 °C overnight. Protein bands were visualized by chemiluminescence (ECL, Forevergen, Guangzhou, China). In the co-immunoprecipitation experiments, cellular lysis was accomplished utilizing a lysis buffer consisting of 140 mM KCl [Sigma-Aldrich, 104936], 3 mM MgCl2 [Sigma-Aldrich, 442615], 1% NP-40 [Sigma-Aldrich, 74385], 20 mM HEPES [Sigma-Aldrich, H23830] (pH 7.4), 1 mM EDTA [Sigma Aldrich, 03609], and 1.5 mM EGTA [Sigma-Aldrich, E4378]. To enhance the efficacy of lysis, 10 mM NaF (Sigma-Aldrich, S7920), 1 mM Na3VO4 (Sigma-Aldrich, 567540), and a protease inhibitor cocktail (Roche, P8849) were incorporated. The lysates were subsequently subjected to centrifugation at 20,000×g for 15 minutes. For immunoprecipitation, 500 µg of protein lysate was allowed to incubate overnight at 4 °C with 2 µg of primary antibody, followed by an additional 4-hour incubation with 50 µl of Rec-Protein G - Sepharose® 4B beads (Thermo Scientific, 101241). To serve as a negative control, an isotype control antibody (Cell Signaling Technology, 3900) was employed. The beads were subjected to three washes in lysis buffer, subsequently incubated for 6 minutes at 95 °C in Laemmli sample buffer (BioRad Laboratories, 1610737), and subjected to SDS-PAGE. The immunoblots were acquired utilizing ChemiDoc Imaging Systems and quantified using the Image Lab software (Bio-Rad Laboratories, Hercules, USA). The experiment was conducted for three times.

### cGAMP stimulation

cGAMP was delivered into cells via permeabilization with digitonin buffer (50 mM HEPES-KOH, pH 7.0, 100 mM KCl, 3 mM MgCl2, 0.1mM DTT, 85 mM Sucrose, 0.2% BSA, 1mM ATP, 10 µg/ml digitonin, along with 10 µg/ml cGAMP (MCE, HY-100564), for 30 min. Following this 30 min incubation, the cells were then incubated in a fresh complete medium supplemented with 10% fetal bovine serum.

### Elisa

ELISA assays for Human IFN-β (QK410), Human CXCL10 (DIP100), Mouse IFN-β (MIFNB0), and Mouse CXCL10 (MCX100), all from R&D Systems, were conducted adhering strictly to the manufacturer's protocols. The conditioned media was harvested from individual cell lines following a 24-hour incubation period. The reported values denote the mean of triplicate measurements taken across a minimum of three separate experiments, ensuring robust biological replication.

### Patients and cancer tissues

This study was approved by the Ethics Committee of Harbin Medical University Cancer Hospital, China. All patients provided written informed consent to participate in this research. The research methodology adhered to the standards outlined in the Helsinki Declaration. From January 2017 to December 2021, a total of 67 HNSCC tissue samples and a subset of normal tissues were collected from Harbin Medical University Cancer Hospital. Two pathologists independently confirmed the diagnosis of HNSCC for all tumor tissues. Patients who had received chemotherapy or radiation therapy were excluded. Overall survival (OS) was defined as the time interval from the date of final diagnosis to the date of death or last follow-up.

### Immunohistochemistry

The expressions of STING on the HNSCC tissue were performed by immunohistochemistry (IHC) staining. Tissue sections were incubated with primary antibody at 4 °C overnight and then incubated with horseradish peroxidase combined with goat anti-rabbit antibody (PV-6001, ZSGB,China) at room temperature for 30 min. Tissue sections were stained using DAB and counterstained with hematoxylin. The results of the experiment were analyzed by two doctors and two pathologists. The rules are as follows: 7, about 50% of tumor cells are strongly stained; 6, about 50% of tumor cells are weakly stained; 5, about 25% of tumor cells are strongly stained; and 4, about 25% of tumor cells showed weak staining; 3,5-25% of tumor cells showed strong staining; 2, about 5-25% of tumor cells showed weak staining; 1,<1% of tumor cells showed low or no staining; 0, no staining was detected in the tumor cells (0%). Samples with a staining score of 0-2 were considered low expression, while samples with a score of 3-7 were considered high expression.

### Mouse tumor model and treatment

The research protocol involving animal studies was approved by the Institutional Review Committee of Xiangya Hospital, Central South University. Male Nude mice and C57BL/6 mice aged 4-6 weeks were obtained from Hunan Slake Jingda Experimental Animal Co., Ltd. and housed at the Department of Animal Science, Central South University. LAMP2A knockout or control Fadu (2×106) or Meer (1×106) cells were subcutaneously injected into the dorsal flank of each nude or C57BL/6 mouse. At the end of four weeks, the mice were euthanized, and the tumors were harvested and weighed. For the evaluation of the combination treatment group. When the tumor size reached 100mm3, 100µg mouse anti-PD-1 antibody (BE0146, Bio X Cell) or IgG control (BE0089, Bio X Cell) was given via intraperitoneal injection (100 mg/injection every 3 days). For the Diclofenac and anti-PD-1 combination treatment, mice were also treated daily with vehicle control (90% polyethylene glycol 400 and 10% DMSO) or Diclofenac (10ug/g every other day, D6899, sigma aldrich) by intraperitoneal injection. When the volume of the inoculated tumor reached 100mm3, the TBK1 antagonist GSK8612 was administered orally at the dose of 5 mg/kg for 10 days and STING inhibitor SN-011 was injected intraperitoneally every 3 days. Tumor sizes were measured every other day. Tumor volume was assessed employing the formula: the product of length and the square of width divided by two, with length denoting the utmost diameter of the tumor and width indicating the minimal diameter. Mice were sacrificed when tumors reached 2000 mm3 or upon ulceration/bleeding.

### Flow cytometry

Employing flow cytometry, we conducted an analysis of the composition of CD8+ T cells derived from peripheral blood and tumor tissue. Tumors were dissected, minced and digested with 1 mg/ml Collagenase/Dispase (Sigma-Aldrich, 10269638001) and 1X DNase I (Qiagen, 79254) at 37°C for 30 minutes. Bloods incubated with red blood cells lysis buffer. Dissociated tumor and peripheral blood mononuclear cells (PBMCs) were filtered through a 70 µm filter to achieve a single-cell suspension before staining for flow cytometry analysis. Cells were stained using Anti-CD8a PE (eBioscience, Cat#12-0081-82, 1:100), anti-CD3e PE-Cyanine7, (eBioscience, Cat#25-0037-42, 1:100), anti-CD3 PerCP-Cyanine5.5, (eBioscience, Cat#45-0037-42, 1:100), anti-PD-1 APC/Cyanine7, (BioLegend, Cat#135224, 1:100), anti-CD69 PerCP-Cyanine5.5, (BioLegend, Cat#134610, 1:100), anti-Ly108 APC, (BioLegend, Cat#134610, 1:100), anti-IFN-γFITC, (BioLegend, Cat#505806, 1:100), and cells were fixed and permeabilized with True-Nuclear™ Transcription Factor Buffer Set (Biolegend, Cat#424401), and dead cells were stained with Ghost Dye™ Violet 510 (Cytek Biosciences, Cat# SKU 13-0870-T100) before fixation and permeabilization and excluded during analysis. Cells were imaged on a BD Biosciences LSRFortessa and analysed with Flowjo. Gating strategies are shown in Supplementary Fig S5C.

### Relationship between risk score and tumor immune microenvironment (TIME)

In this study, we selected cellular estimates37, 22 immune cells of Cibersort38 and TCR richness39 as TIME-relevant molecular signatures. “IOBR” R package 40 was utilized to quantify the scores of all TIME-relevant molecular signatures.

### Statistical analysis

All the experiments were repeated at least three times. The data were analyzed using SPSS 20.0, R software 4.0.3 (Institute for Statistics and Mathematics, Vienna, Austria) and GraphPad Prism 8. The P-value <0.05 was considered to be significant.

## Supplementary Material

Supplementary figures and tables.

## Abbreviations

CMA: chaperone-mediated autophagy
HNSCC: head and neck squamous cell carcinoma
MDSCs: myeloid-derived suppressor cells
HSPA8: heat shock protein family A member 8
ICB: immune checkpoint blockade
PD-1 mAb: pd-1 monoclonal antibody
NSAIDs: non-steroidal anti-inflammatory drugs
mIHC: multiplex immunohistochemistry
DOK: dysplastic oral keratinocyte
IB: immunoblot
Ub-conj: ubiquitin-conjugated proteins
