# Supplementary material for: Enhanced therapeutic efficacy of anti-PD-1 blockade by targeting LAMP2A inhibits lysosomal degradation of STING and TBK1
Source: Theranostics. 2025 Feb 18;15(7):3207–22. doi: 10.7150/thno.103426 (PMC11898276; doi:10.7150/thno.103426)
Supplement: Supplementary file 1 — Supplementary figures and tables. [file thnov15p3207s1.pdf]

Table S1: Correlation between LAMP2A and clinical indicators by univariate COX analysis

**Characteristic**	**Total population**	**LAMP2A-high**	**LAMP2A-low**	**P**
Total	67(100)	47(70.1)	20(29.9)	
Age at diagnosis				
＜60	39(58.2)	30(44.7)	9(13.5)	0.075
≥60	28(41.8)	17(25.4)	11(16.4)	
T stage				
T1	23(34.3)	21(44.7)	2(10.0)	0.014
T2-3	44(65.7)	26(55.3)	18(90.0)	
Lymph node metastasis				
Negative	34(50.7)	33(70.2)	1(5.0)	<0.001
Positive	33(49.3)	14(29.8)	19(95.0)	
KI67 expression				
Low	42(62.7)	39(83.0)	3(15.0)	<0.001
High	25(37.3)	8(17.0)	17(85.0)	
Differentiation				
Well	31(46.3)	27(57.4)	4(20.0)	0.005
Poor	36(53.7)	20(42.6)	16(80.0)	
Vascular invasion				
Negative	41(61.2)	31(66.0)	10(50.0)	0.220
Positive	26(38.8)	16(34.0)	10(50.0)	

Table S2:All plasmid sequences

Type	Target	Sequence
siRNA	siNC	5'-AUGGCAUCAUAAGCUGCACAC-3'
siRNA	LAMP2A#1	5'-TGGCAGGAGTACTTATTCTAG-3'
siRNA	LAMP2A#2	5'-CATCATGCTGGATATGAGCAA-3'
siRNA	LAMP2A#3	5′-GGUCUCAAGCGCCAUCAUATT-3′
siRNA	ATG5	5′-CAUCUGAGCUACCCGGAUA-3′
siRNA	ATG7	5′-CTTGATCAGTACGAGCGAGAA-3′
shRNA	shNC#1 Forward	CCGGCAACAAGATGAAGAGCACCAACTCGAGTTGGT GCTCTTCATCTTGTTGTTTTTG
shRNA	shNC#1 Forward	AATTCAAAAACAACAAGATGAAGAGCACCAACTCGA GAAGGTGCTCTTCATCTTGTTG
shRNA	shLAMP2A#1 Forward	CCGGTCTAGTGTTGCTGGCTTATTTCTCGAGAAATAA GCCAGCAACACTAGATTTTTG
shRNA	shLAMP2A#1 Reverse	AATTCAAAAATCTAGTGTTGCTGGCTTATTTCTCGAG AAATAAGCCAGCAACACTAGA
shRNA	shLAMP2A#2 Forward	CCGGAGCTCTGGGAGGAGTACTTATCTCGAGATAAGT ACTCCTCCCAGAGCTTTTTTG
shRNA	shLAMP2A#2 Reverse	AATTCAAAAAAGCTCTGGGAGGAGTACTTATCTCGAG ATAAGTACTCCTCCCAGAGCT
sgSTING	H-STING	GGATGTTCAGTGCCTGCGAG
sgTBK1	H-TBK1	CATAAGCTTCCTTCGTCCAG
sgsting	M-sting	TACCTTGGTAGACAATGAGG
sgtbk1	M-tbk1	CGGGAACAACTCAATACCGT
sgScramble	Scramble	ATTGTTCGACCGTCTACGGG

Table S3: The sequences of PCR primers

Gene	Forward	Reverse
H-STING	GAGTGTGTGGAGTCCTGCTC	CTGGAGTGGGGCATCTTCTG
H-TBK1	GGAGACCCGGCTGGTATAA	GGAGACCCGGCTGGTATAA
H-IFNB1	CATTACCTGAAGGCCAAGGA	CAATTGTCCAGTCCCAGAGG
H-CXCL10	GTGGCATTCAAGGAGTACCTC	TGATGGCCTTCGATTCTGGATT
H-ACTIN	GGCTGTATTCCCCTCCATCG	CCAGTTGGTAACAATGCCATGT
M-ifnb1	ATGAGTGGTGGTTGCAGGC	TGACCTTTCAAATGCAGTAGATTCA
M-cxcl10	ATGACGGGCCAGTGAGAATG	TCAACACGTGGGCAGGATAG
M-actin	AGTGTGACGTTGACATCCGT	GCAGCTCAGTAACAGTCCGC

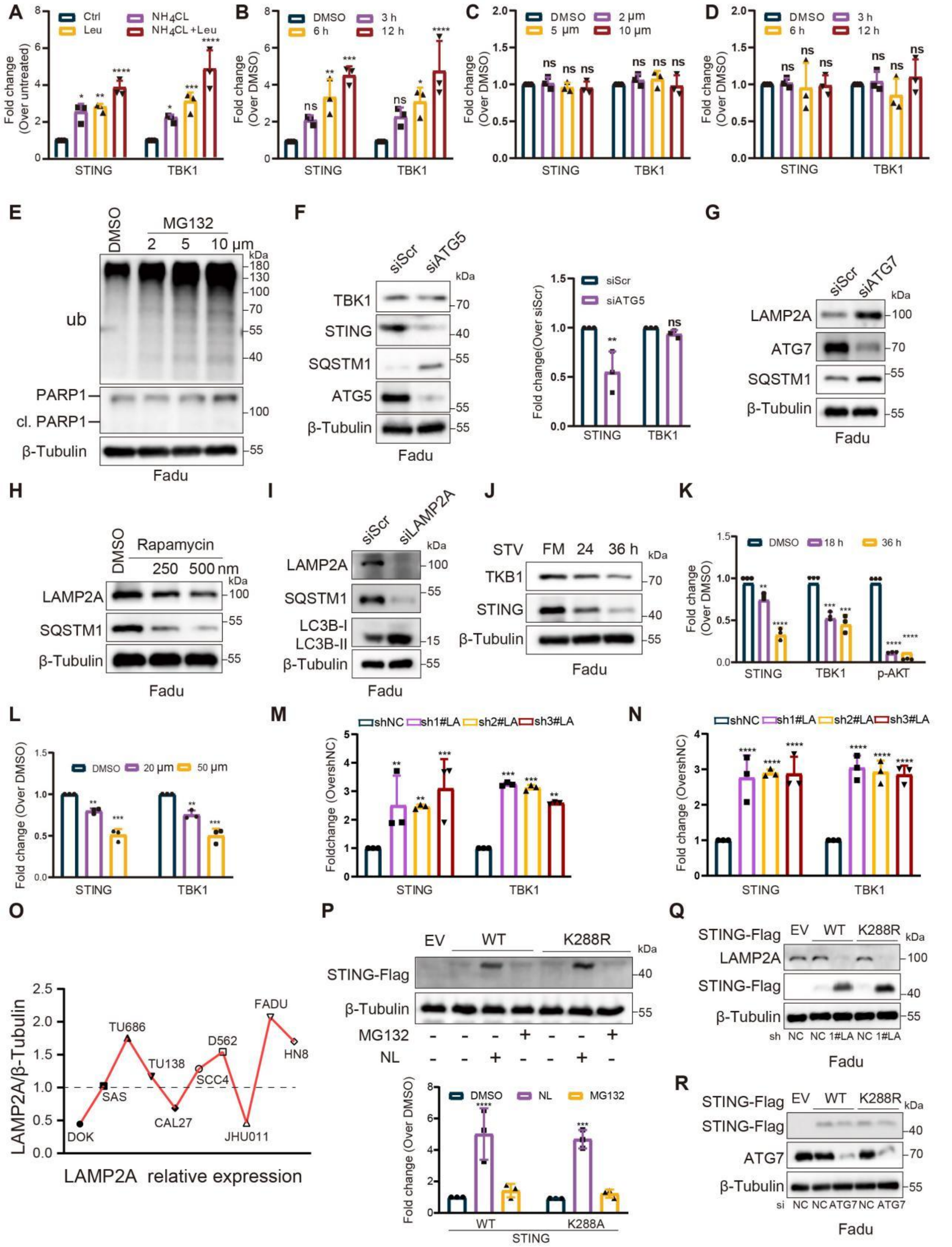

Figure S1. **The modulation of CMA exerts an impact on STING and TBK1 protein levels. (A)** Western blot analysis of ubiquitin-conjugated proteins (Ub-conj) and apoptosis marker PARP1 in Fadu cells treated for 6 h with the indicated concentrations of MG132. **(B)** Knockdown of ATG5, a macroautophagy inhibitory molecule, in Fadu cells did not result in the accumulation of STING and TBK1. **(C)** Silencing of ATG7, a macroautophagy inhibitory molecule, increases LAMP2A in Fadu cells. SQSTM1 accumulation is shown as a positive control. **(D)** Treatment of Fadu cells for 24 h with the indicated concentrations of the macroautophagy-inducing drug rapamycin reduces the levels of LAMP2A. SQSTM1 reduction is shown as a positive control. **(E)** Silencing

of LAMP2A causes a moderate decrease of SQSTM1 and an increase of MAP1LC3-II, compatible with an increased autophagic flux. Related to Figure 1. **(F)** Prolonged starvation reduces protein levels of STING and TBK1 in Fadu cells. (G) The baseline expression levels of LAMP2A in HNSCC cell lines. (H, I) Knockdown of LAMP2A (E) but not ATG7 (C) increases the expression of both STING WT and STING K288R in Fadu cells.

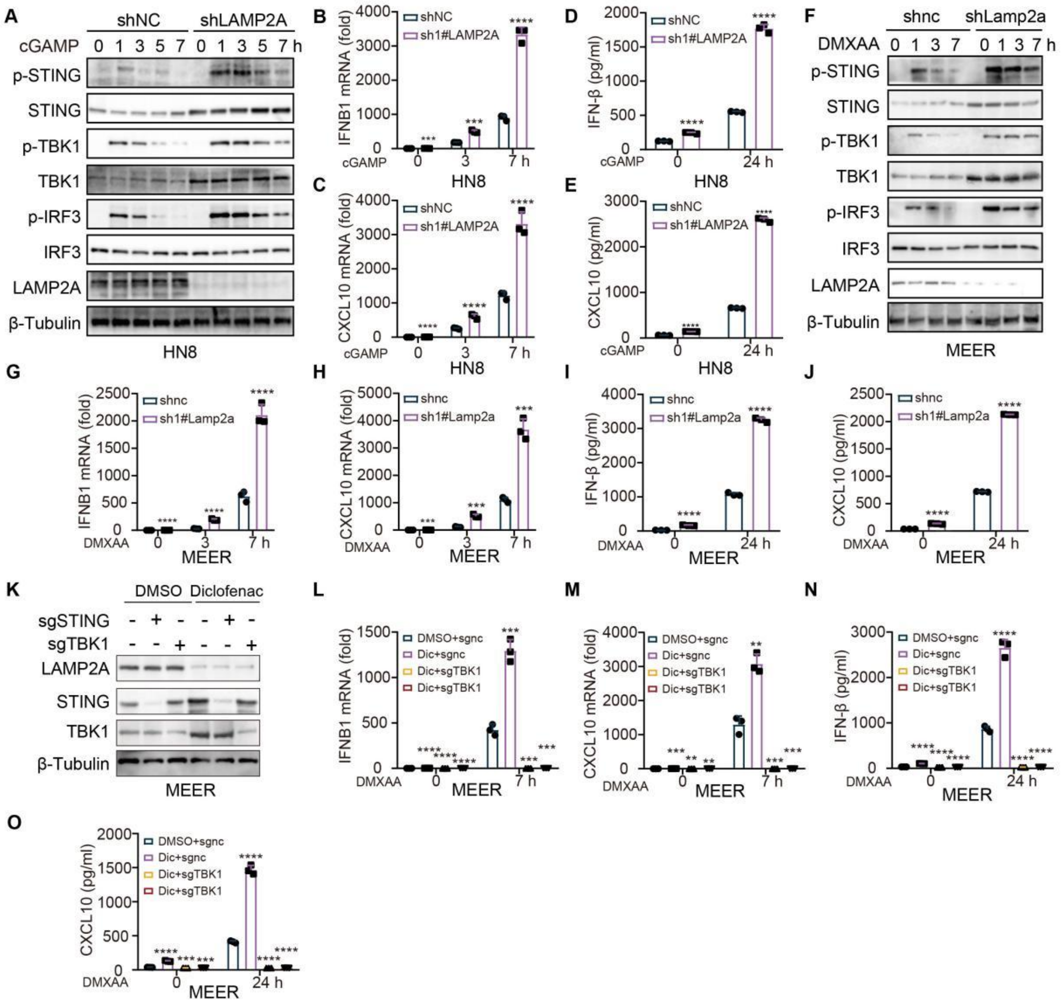

Figure S2. LAMP2A deficiency chronically activates STING-mediated type I IFN signalling. **(A)** IB of the indicated proteins in HN8 cells, which treated with the cGAMP (10μg/ml) for 0, 3, 6, 9 or 12 h. **(B-E)** Quantitative polymerase chain reaction (PCR) analysis of IFNB1 (B) and CXCL10 (C) mRNA and ELISA of human IFN-β (D) and CXCL10 (E) levels in conditioned medium derived from HN8 cells which treated with the indicated treatment. **(F)** IB of the indicated proteins in MEER cells after stimulation with DMXAA (the STING agonist, 50 μg/ml). **(G-J)** qPCR analysis of Ifnb1 (G) and Cxcl10 (H) mRNA and ELISA of Ifn-β (I) and Cxcl10 (J) levels with the indicated treatment. (K) IB was employed to confirm the efficacy of both STING and TBK1 knockouts, as well as to ascertain the effectiveness of Diclofenac treatment. **(L-O)** qPCR analysis of Ifnb1(L) and Cxcl10(M) expression for the indicated times and ELISA analysis of Ifn-β (N) and Cxcl10 (O) production in the cell culture supernatants after stimulated with

DMXAA (50 μg/ml). Data are representative of three independent experiments with similar results means ± SD. NS, not significant, *P < 0.05, **P < 0.01, and ***P < 0.001, ****P < 0.0001.

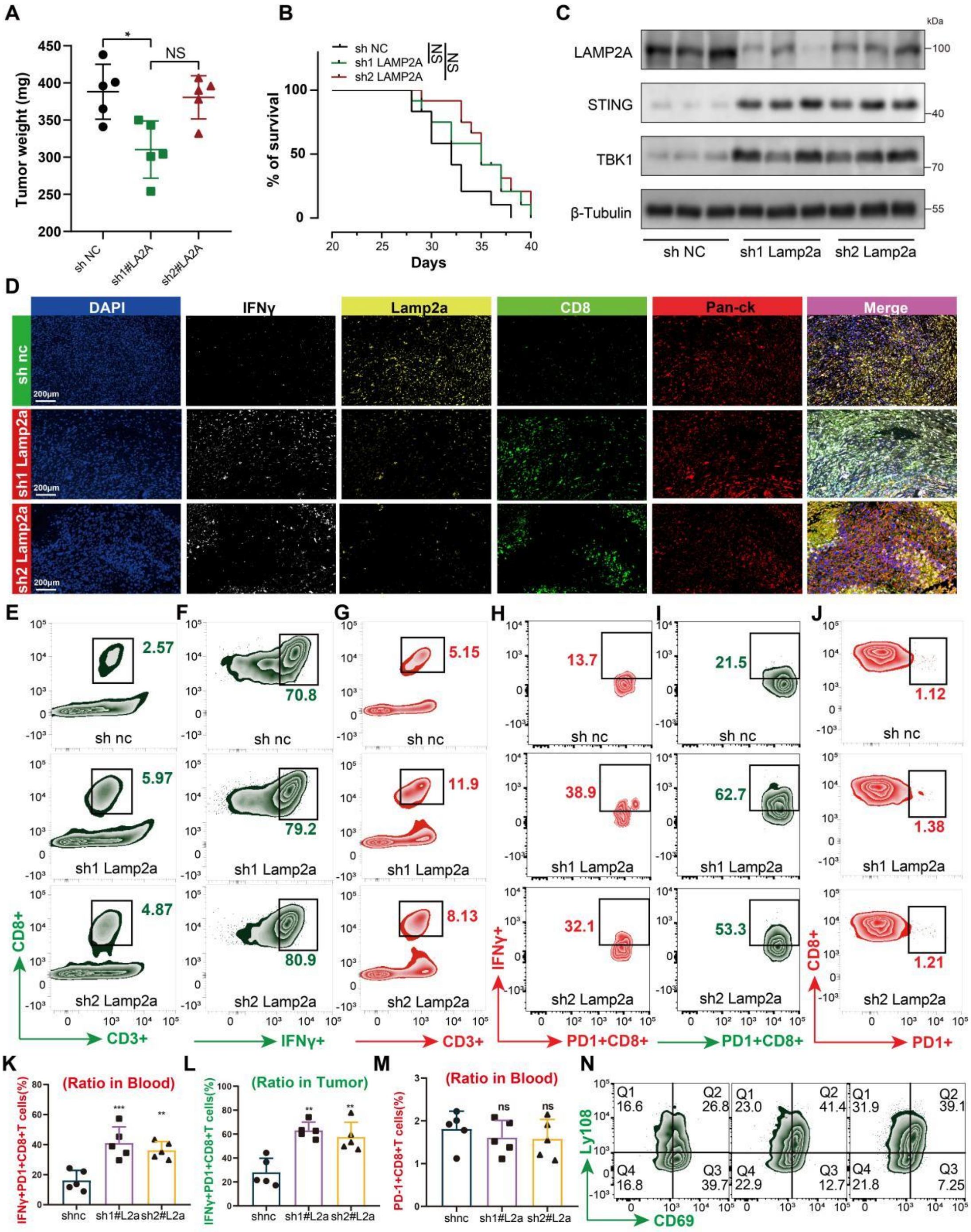

Figure S3. **LAMP2A inhibit anti-tumor immunity. (A,B)** Summary of tumor weight data of Fadu tumors harvested after euthanizing the mice(A) and Kaplan-Meier survival curves for each group(B). **(C)** Differences in the expression of LAMP2A, STING, and TBK1 in tumor tissues among different groups. **(D)** mIHC staining of IFNγ, Lamp2a, CD8 and Pan-ck of shLamp2a or shNC Meer xenografts. **(E, F)** FACS of CD8+ in CD3+ cells and IFNγ+ in CD8+ cells TILs from shLamp2a or shNC Meer xenografts, correlated with Figures 5H, I. **(G)** FACS of CD8+ in CD3+ cells blood from shLamp2a or shNC Meer xenografts, correlated with Figures 5J. **(H, I)** FACS analysis of IFNγ+PD-1+CD8+ cells in the peripheral blood (H) and tumor tissue (I) of

xenotransplanted mice bearing shLamp2a or shnc, correlated with Figures S4K, L. **(J)** FACS of PD-1+ in CD8+ cells from peripheral blood of xenotransplanted mice originating from shLamp2a or shnc, correlated with Figures S4M. **(N)** FACS of LY108(-/+) CD69(-/+) cells in CD3+ TILs from shLamp2a or shnc Meer xenografts. log-rank test for survival comparison. Means ± SD. NS, not significant, *P < 0.05, **P < 0.01, and ***P < 0.001, ****P < 0.0001.

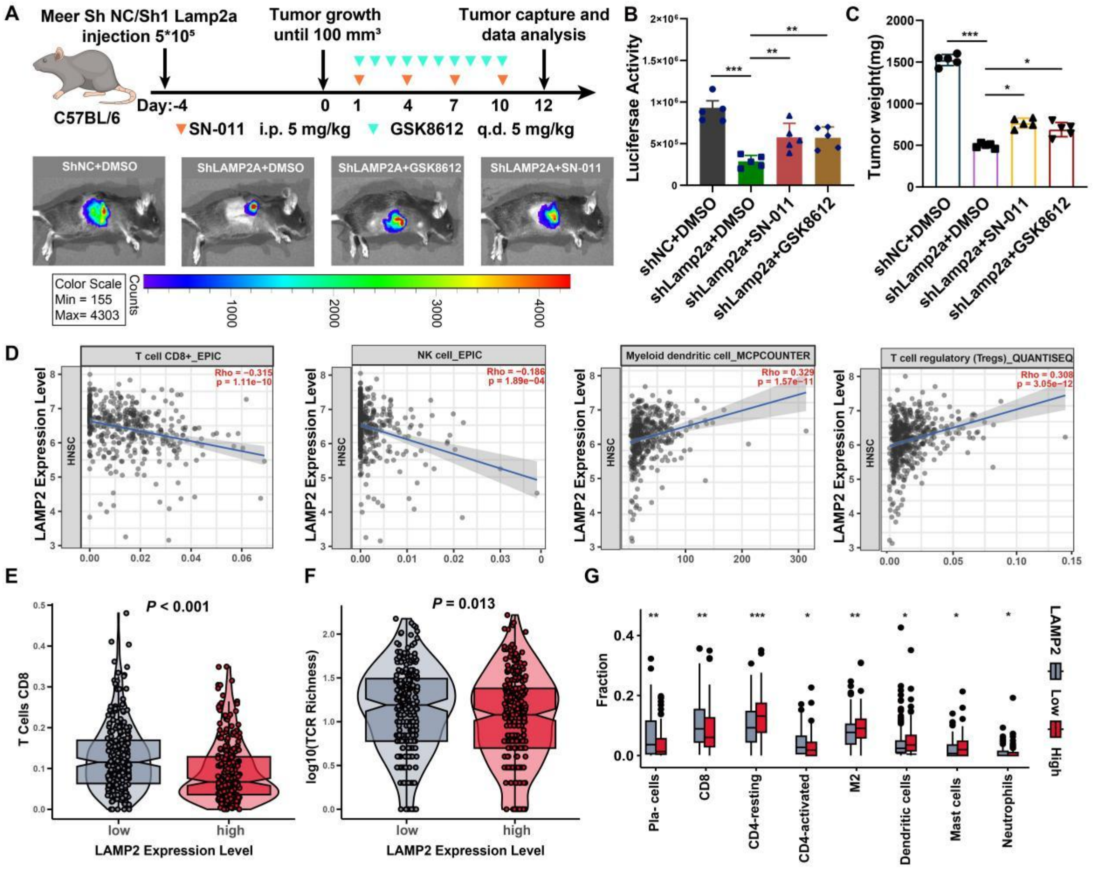

Figure S4. **Blocking STING-TBK1 reversed the anti-tumor effect of LAMP2A (A)** schematic view of the treatment plan and subcutaneous tumor images of the Meer cells. **(B, C)** Summary of luciferase activity (B) and tumor weight data (C) of meer tumors harvested after euthanizing the mice. **(D)** Conducting an analysis using the TCGA dataset to examine the correlation between LAMP2 expression levels and the abundance of CD8+ T cells, NK cells, myeloid dendritic cells, and Tregs. **(E, F)** Comparison of CD8 and TCR richness between the high and low LAMP2 expression groups patients. **(G)** Boxplots depicting the CIBERSORT scores of 8 immune cells of the high LAMP2 patients compared to low patients.Means ± SD. NS, not significant, *p < 0.05, **p < 0.01, ***p < 0.001 vs. control.

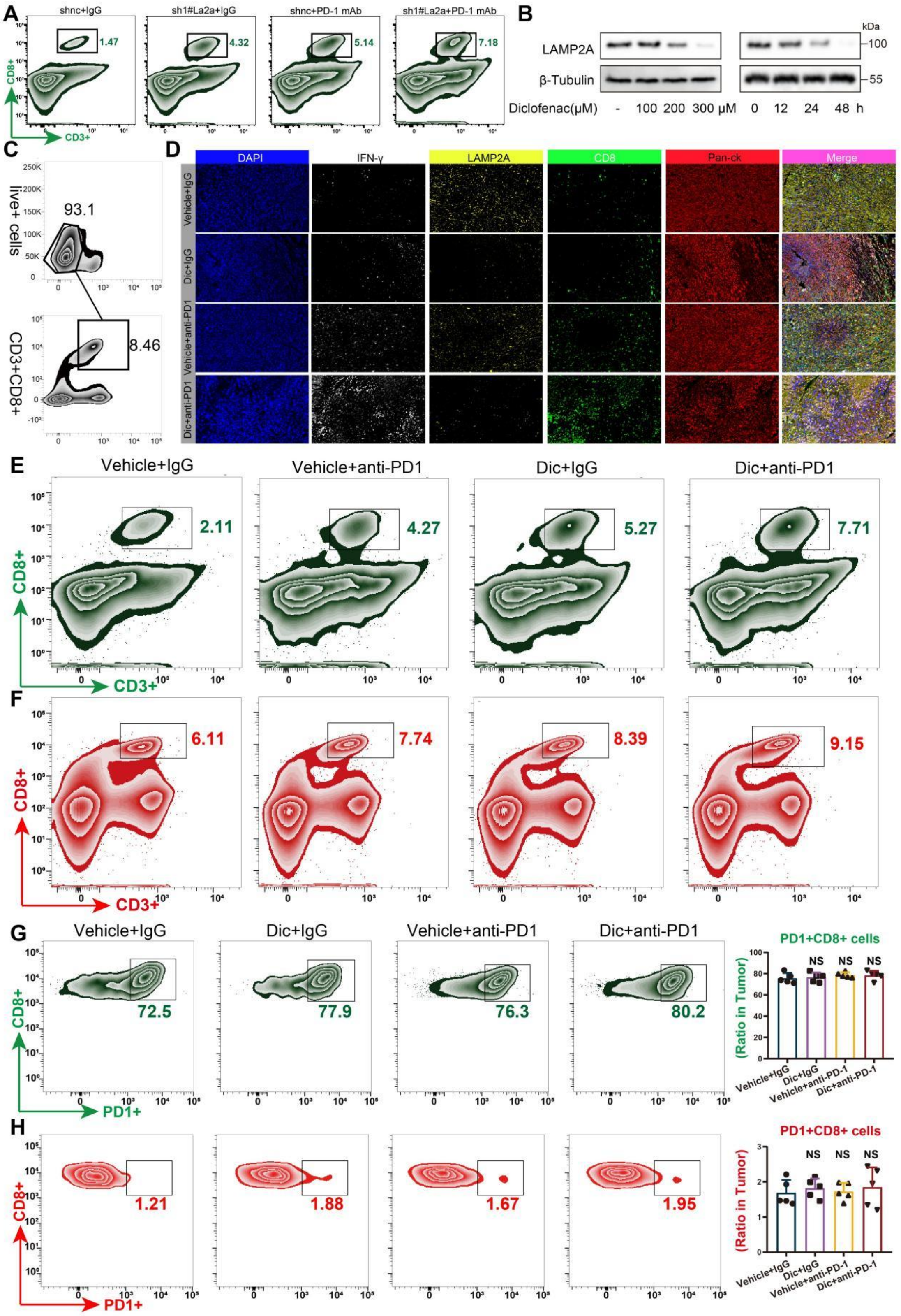

Figure S5. **The inhibitor of LAMP2A, diclofenac, synergistically enhances the anticancer effect of PD-1. (A)** FACS of CD8+ in CD3+ cells from TILs of different groups Meer xenografts, correlated with Figures 6D. **(B)** Western blot analysis of LAMP2A expression levels in Meer cells after 24 hours of treatment with varying concentrations of diclofenac and following treatment at different time intervals. **(C)** Flow cytometry coil-gate strategy. **(D, E)** FACS of CD8+ in CD3+

cells from TILs of heterologous Meer mouse transplant tumors (D) and blood samples (E) in different groups. **(F, G)** FACS of PD-1+CD8+ in CD3+ TILs from from heterologous Meer mouse transplant tumors (F) and blood samples (G) in different groups. Means ± SD. NS, not significant, *p < 0.05, **p < 0.01, ***p < 0.001 vs. control.

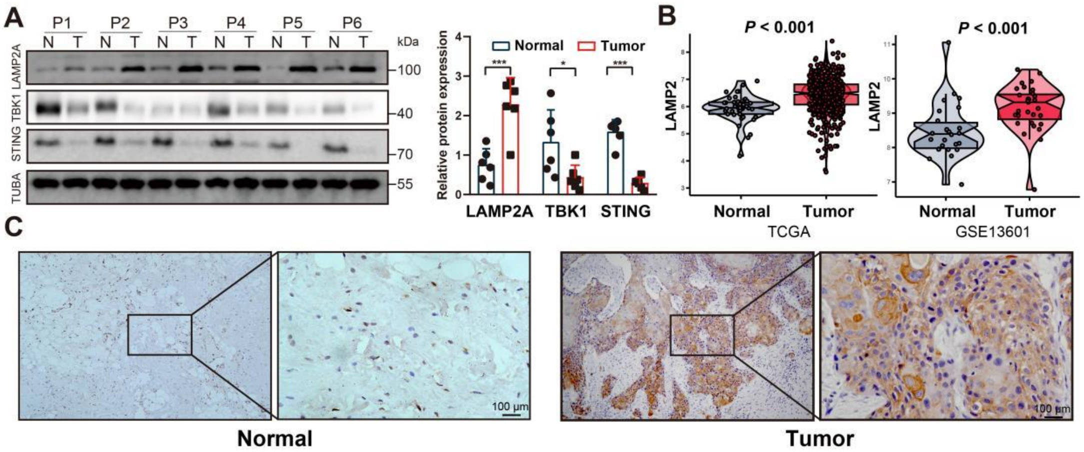

Figure S6. **LAMP2A exhibits high expression levels within tumor tissues. (A)** The expression levels of LAMP2A in clinical samples from both tumor and adjacent non-tumor tissues. **(B)** The expression levels of LAMP2 in various datasets. **(C)** Immunohistochemical representative images of tumor and adjacent normal tissues from clinical samples.Means ± SD. NS, not significant, *p < 0.05, **p < 0.01, ***p < 0.001 vs. control.
